# Measuring the monetization strategies of websites with application to pro- and anti-vaccine communities

**DOI:** 10.1038/s41598-023-43061-6

**Published:** 2023-09-25

**Authors:** David A. Broniatowski, Kevin T. Greene, Nilima Pisharody, Daniel J. Rogers, Jacob N. Shapiro

**Affiliations:** 1 The George Washington University, Washington, USA; 2 Princeton University, Princeton, USA; 3 The Global Disinformation Index, London, UK

**Keywords:** Computational science, Public health

## Abstract

Anti-vaccine content and other kinds of misinformation are hypothesized to be more heavily monetized than other kinds of online content. We test this hypothesis by applying several novel and scalable measures of website monetization strategies to more than 400,000 links shared by 261 anti-vaccine Facebook pages and 190 pro-vaccine ones. Contrary to expectations, websites promoted in pro-vaccine venues do more to monetize attention than those promoted in anti-vaccine venues. This is a consequence of how intensely monetized news websites are—pro-vaccine venues share more links to news. The specific news sites shared by anti-vaccine venues are rated less credible by fact-checking organizations, but we find little substantive difference in their monetization strategies. These results emphasize the need to interpret measures of monetization within the context of the broader “attention economy”.

## Introduction

Is the economy of online misinformation different from that of the broader online attention economy? To answer such questions we need scalable measures of how websites seek to monetize attention that enable comparisons between content promoted by different communities. Such measures are particularly critical for researchers interested in the financial motivations for spreading misinformation^1–4^.

Recent research suggests that the “attention economy” offers new opportunities for individuals or organizations to profit off of online information^5–7^, misinformation through ads^8^, and selling low-quality products^9–13^. Some venues appear to have generated significant social media attention for fake content primarily to earn revenue from engagement^14^, while others seem to have promoted low-information coronavirus-related content mainly to solicit donations^12,13,15^. This body of research has drawn attention to the possibility that legitimate sources^16,17^ are inadvertently facilitating the flow of funds to misinformation disseminators, and to calls for closer scrutiny for advertisements and infrastructure providers^18–20^.

Monetization strategies are especially important to understand in the context of the discourse on vaccines where there is significant concern that financially motivated anti-vaccine websites are contributing to increasing vaccine hesitancy^21–24^. To date, there is only a single attempt to measure the broader monetization strategies of anti-vaccine sites. Herasimenka et al.^25^ manually identify anti-vaccine actors with websites and measure the presence or absence of four specific approaches to monetization (donations, sales, advertising, and membership dues). They find that these sites mostly use a hybrid monetization strategy, relying on monetizing both attention and collecting dues from supporters.

Although^25^ expands productively on past work by evaluating the monetization strategies of 59 sites they label as primarily anti-vaccine, the selection criteria for potential anti-vaccine sites are somewhat arbitrary. Their sample is based on sites shared by Twitter users in 2016 and 2018^26^, but the process of selecting anti-vaccine sites from 2020 to 2021 is unclear. Without a more generalizable sample selection strategy, it is hard to judge how much their findings support broader claims about anti-vaccine sites.

We introduce a new approach to measuring efforts to profit from online content and apply it to anti-vaccine discourse. Our approach breaks new ground on five dimensions.

First, we compare sites promoted or managed by anti-vaccine actors to those employed by other actors.

Second, our sampling strategy is designed to reduce sampling bias associated with keyword selection. To do so, we draw upon a two-stage website sampling process. In stage one we annotate more than 400 vaccine-related Facebook groups/pages (referred to as venues or actors throughout the text) as either pro or anti-vaccine (Cohen’s k = 0.88, 95% CI 0.85–0.92)^27^. In stage two we extract each domain shared in these venues from November 2019 to August 2021. These shared links represent the revealed preferences of vaccine-related venues and shed light on the information sources which support their positions. This enables us to evaluate whether anti-vaccine actors rely on more highly monetized sources than others or if efforts to profit from ads^8^ and selling products^9–11^ are broader features of the attention economy^5^. Specifically, we compare the monetization intensity of domains shared in anti-vaccine Facebook venues to those shared in pro-vaccine Facebook venues. This allows us to evaluate differences in the monetization strategies used by sites cited by anti-vaccine actors vs. pro-vaccine actors, which offers evidence on whether the anti-vaccine discourse is uniquely monetized.

Third, we analyze a larger sample of sites than has been used in prior work, which has either analyzed single sites^22,24^, or a small sample of prominent sites^21,23^. In total, our data from more than 400,000 URLs represent more than 2600 websites.

Fourth, our work is scalable. Current measures of monetization are constructed by human coders manually annotating the presence or absence of specific monetization strategies. As the number of sites to evaluate increases, this approach becomes untenable. Further, as new websites emerge, past efforts would have to be duplicated to remain relevant, requiring considerable time and effort. This shortcoming is particularly notable for studying the monetization of misinformation, where prior work suggests sites will rapidly rise and fall in popularity as new sites take their place^28^.

Fifth our approach is high fidelity. Past efforts have treated monetization strategies as binary measures, capturing only the presence or absence of a given monetization strategy. Such measures miss differences in how much emphasis sites place on making money, e.g., between sites that host one or two ads, and those which host many more. Further, there may be significant variation in the amount of page space that ads occupy, which current measures do not capture.

Specifically, we introduce four measures of the monetization strategies of web domains (described in detail below):

- *Ad density* The ratio of ad space to text content on the page;
- *Ad count* The number of ads on a page;
- *Donation count* The number of donation solicitations on a page;
- *Outbound links* The number of point-of-reference dependent links on a page.

Using computational tools to extract the required information within the HTML of webpages in this manner facilitates large-scale data collection and replication.

We find that fully 98% of the domains in our sample display some form of monetization strategy. Overall, embedded ads are the most common strategy, with 96% of domains dedicating at least some portion of their site to ads. Contrary to expectations, pro-vaccine actors tend to share more heavily monetized sources than anti-vaccine actors. This is largely because pro-vaccine venues share more links to news than their anti-vaccine counterparts, and news websites are more highly monetized overall. We find little evidence of differences in monetization between the news sites shared by anti-vaccine venues and those shared by pro-vaccine venues, though the former tended to be rated lower by news rating organizations.

We note that our results do have one important limitation. While our approach illuminates differences in the monetization strategies between information sources cited by these two communities, it does not capture how much money those sources actually bring in from the strategies they use. Thus we cannot say whether there is a difference in the amount of money raised by pro- vs. anti-vaccine websites.

## Results

**Table 1:** The usage of monetization strategies in sites shared by vaccine-related Facebook venues.

Measure type	Num. of sites	Percentage
HTML measures		
Ad count	1588	65.22
Donation count	1978	81.23
URL measures		
Ad occupancy	2353	98.45
Outbound links	2141	89.96

We present the count and percentage of domains that display evidence of a given monetization strategy. Facebook venues from^27^.

We first present the usage of monetization strategies across sites shared by both anti and pro-vaccine actors (Table 1). Across the more than 2400 domains we evaluated nearly all featured some form of monetization strategy. Embedded ads were the most commonly employed monetization strategy; over 98% of sites dedicate at least some of their page space to ads. This result contrasts with the findings of^25^. This may be due in part to^25^ counting only banner ads, potentially missing ads embedded within the text of articles or popup ads. We observe a considerably lower percentage of sites displaying ads based on our Ad Count measure. This is primarily because the Ad Count measure does not capture pop-up ads. We similarly observed that most sites in our sample solicit donations and embed outbound links to other domains. This again differs from^25^ who find that 58% of sites appeal for donations. Overall, the domains in our sample are likely to use some monetization strategy, with 95% using more than one.

**Figure 1:**
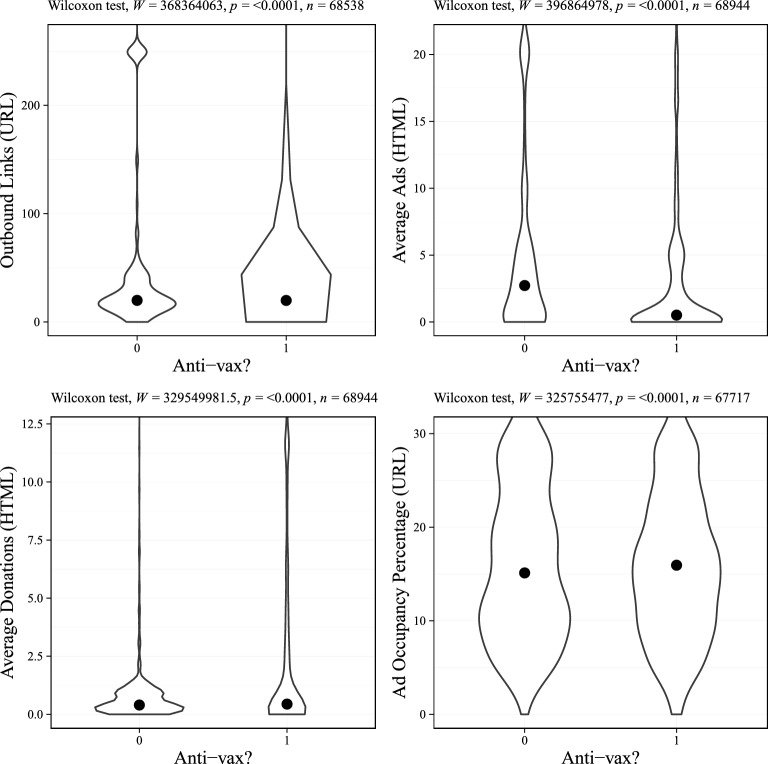
The monetization of sites shared in anti and pro-vaccine Facebook venues. Each subplot captures a different monetization measure. Facebook venues from^27^.

To compare the overall differences in the monetization of sites between anti and pro-vaccine actors, we calculate our four measures across each of the domains shared by pro and anti-vaccine actors. We report violin plots of the distribution of each measure for domains shared in anti and pro-vaccine venues in Fig. 1. First, we find a statistically significant and substantive difference in the average number of ads on sites shared by pro- and anti-vaccine groups. Domains shared in anti-vaccine venues have a median of 0.51 ads compared to 2.73 for pro-vaccine. The results of a Mann–Whitney *U* test indicate a statistically significant difference between the groups (U = 396,864,978, p ≤ 0.0001, n = 68,944). Second, across our other measures, we find little evidence that sites shared in anti-vaccine venues are more heavily monetized than those in pro-vaccine venues. For our Ad Occupancy and Donation measures, we find that there is a statistically significant difference in the monetization of sites between anti- and pro-vaccine venues, but the magnitude of the difference is very small (anti-vaccine = 0.40, pro-vaccine = 0.44; anti-vaccine = 15.10, pro-vaccine = 15.90). The results of a Mann–Whitney *U* test (U = 368,364,063, p ≤ 0.0001, n = 68,538) and violin plots indicate more Outbound Links for sites shared in anti-vaccine groups.

**Figure 2:**
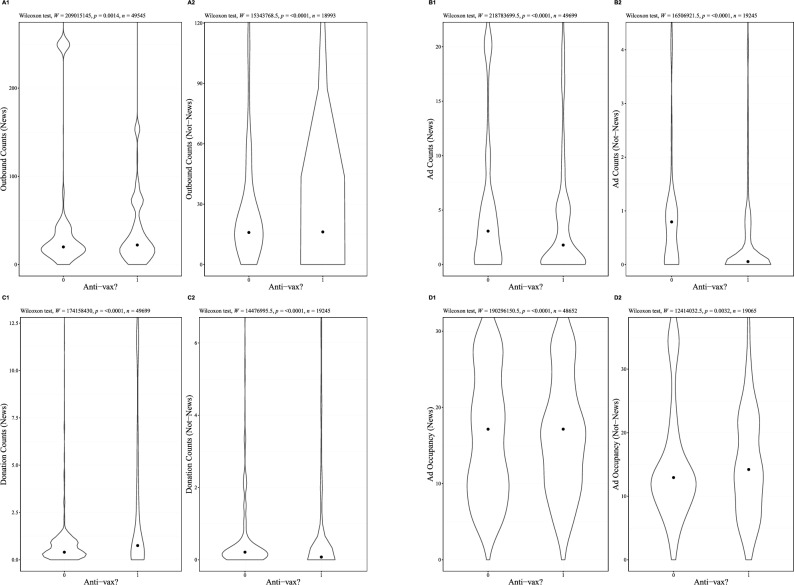
The monetization of sites shared in anti and pro-vaccine Facebook venues. Each measure shows the monetization for news and non-news sites. News/non-news based on inclusion in^29^. Facebook venues from^27^.

To further characterize differences in the monetization strategies of anti- and pro-vaccine actors, we evaluate the types of sites shared by these venues. We divide the domains in our study into news sites and non-news sites. This distinction is interesting as online news sites aim to both monetize attention and provide information, creating a trade-off between content and monetization strategies. We consider a domain to be a news site if its quality has been assessed in^29^. They report measures of the quality of information for thousands of domains and show a high correlation between measures. In Fig. 2 we reproduce the results presented in Fig. 1, now distinguishing between news and non-news sites. Three findings emerge. First, for each measure other than Outbound Links, news sites are more heavily monetized than non-news sites (e.g. Fig. 2, D1 vs. D2). Second, with the exception of Ad Counts, the news sites shared in anti- and pro-vaccine venues have similar levels of monetization (e.g. Fig. 2, A1 vs. A2). Third, while news sites are often more heavily monetized, aside from Outbound Links, there are no major differences between the groups for news and non-news sites. This illustrates how pro- and anti-vaccine venues arrive at similar levels of monetization through different channels. Pro-vaccine venues regularly share links to highly monetized news sites, while anti-vaccine venues share heavily monetized non-news sites. Roughly 88% of the links shared in pro-vaccine venues are to news sites, compared to roughly 67% for anti-vaccine venues. The results also suggest that monetization is not a distinguishing feature of anti-vaccine content but rather ubiquitous in online news.

**Figure 3:**
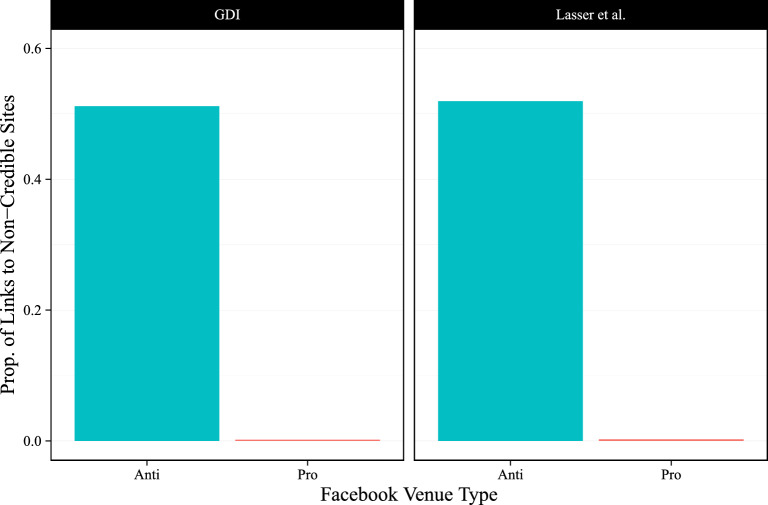
The proportion of non-credible news sites shared in anti- and pro-vaccine Facebook venues. News/non-news classifications are based on inclusion in^29^. Source credibility ratings are from the Global Disinformation Index (GDI) and^30^. Facebook venues are from^27^.

**Figure 4:**
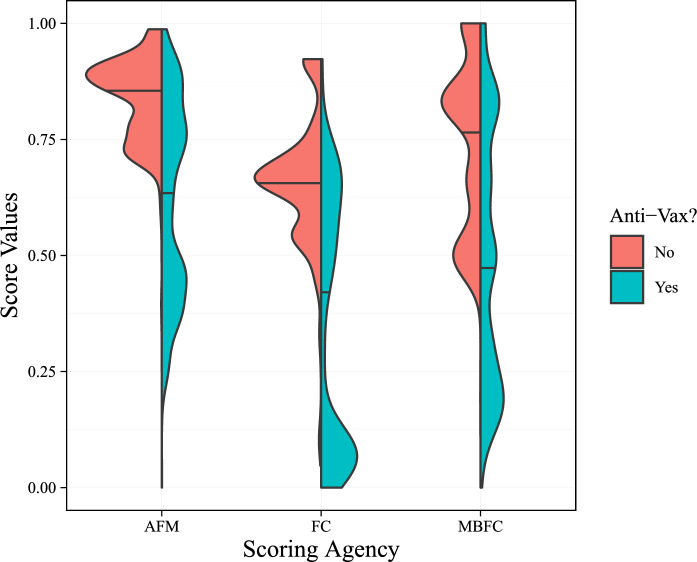
The credibility of news sites shared in anti- and pro-vaccine Facebook venues. News/non-news classifications are based on inclusion^29^. Source credibility ratings are from Ad Fontes Media (AFM), professional fact-checkers (FC), Media Bias/Fact-Check (MBFC), using measures from^29^. Facebook venues are from^27^. Horizontal lines indicate median values for each group.

Links shared in both pro- and anti-vaccine venues frequently cite news sources that feature similar levels of monetization. However, these venues may share different types of news sites to advance their positions. To address this possibility, we examine the extent to which pro- and anti-vaccine Facebook actors share links to information sources rated as unreliable by different organizations. We first use binary measures of site credibility drawn from the Global Disinformation Index’s (GDI) Dynamic Exclusion List and^30^. We find that anti-vaccine Facebook venues share a much higher proportion of links to unreliable information sources (Fig. 3). Roughly 0.2% of the domains shared by pro-vaccine venues were rated as unreliable compared to roughly 50% for anti-vaccine venues. Second, we utilize three continuous measures of site credibility drawn from^29^ who standardized scales across different rating organizations. Across these measures, we find large differences in the credibility distribution of sites shared in anti- and pro-vaccine venues (Fig. 4). The median credibility rating for sites shared by pro-vaccine actors is consistently higher than those shared by anti-vaccine actors, and the distributional differences are even starker. While pro-vaccine venues primarily share news from highly-rated sources, the distribution of credibility ratings for anti-vaccine sites feature more density in the lower tails, indicating that these venues share content from both the highest and lowest rated sources. These results suggest that high monetization is not necessarily a marker of unreliable content. We see plenty of highly-rated sites that intensively monetize their content, in line with the broader incentives of the attention economy^5,7^.

## Discussion

We introduce a novel, scalable and replicable set of measures of the monetization strategies of online discourse and apply it to websites cited by pro- or anti-vaccine social media venues. Nearly all 4619 domains in our sample of URLs linked to by more than 400 vaccine-related Facebook groups/pages display some form of monetization. In contrast to^25^, we find that embedded ads are the most commonly used monetization strategy, present in roughly 98% of domains. Rather than anti-vaccine actors relying on more heavily monetized domains, both sides of the vaccine debate are highly monetized. Interestingly, for some measures, pro-vaccine actors share more heavily monetized sources than anti-vaccine actors. This may be due to the financial incentives to profit from attention^5^ resulting in even high-quality media sites being heavily monetized. Despite the similar overall monetization, anti- and pro-vaccine actors rely on different sets of highly monetized sites. Anti-vaccine venues frequently share highly monetized non-news sources and low-credibility news sites, while pro-vaccine venues more often share credible news sites.

Our work has several implications for research on the monetization of content generally and specifically on the vaccine discourse. First, understanding if anti-vaccine venues are more heavily monetized or rely on distinct strategies requires making comparisons between anti- and pro-vaccine venues. Past work has tended to evaluate only the monetization strategies of anti-vaccine actors. Second, given our findings that both sides of the vaccine debate are highly monetized it is worth further investigating the monetization strategies of pro-vaccine sites. Several sites have sold bogus COVID-19 test kits and used interest in vaccination to collect medical and financial information^31^. There are also numerous examples of sites profiting through posting affiliate links to pro-vaccine merchandise^32,33^.

Our work also has implications for understanding the monetization of online information more generally. The measures we introduce can be readily applied to a variety of additional research questions and capture both strategies used as well as how much space sites dedicate to trying to make money off of users. For instance, researchers might investigate if more highly monetized sites pushed more pro-Russian narratives after the invasion of Ukraine or if there are differences in the monetization intensity of sources shared across political parties.

Some limitations of our study should be noted. First, as with all measures of dynamic processes, our results are a snapshot of a site’s monetization at that particular point in time. However, because our code can be easily executed at additional time points, this limitation can be mitigated. Further, this ability to easily reassess a site better allows a researcher to evaluate changes in the monetization strategies of domains, which may provide new insights into the impacts of demonetization efforts.

Second, while approaches drawing on website presentation reveal underlying monetization strategies, they are not necessarily informative about how these strategies are actualized by sites. A site might be highly monetized in the sense of dedicating a great deal of space to advertising or having many subscription links, without earning significant income. Similarly, while our approach illuminates differences in the monetization strategies between information sources cited by these two communities, we cannot say whether there is a difference in the amount of money raised by pro- vs. anti-vaccine websites.

Third, and relatedly, while sites shared by anti- and pro-vaccine venues may be similarly monetized, that does not mean they generate the same revenue per ad. Ad platforms may be less inclined to allow anti-vaccine sites to host ads as groups such as the Global Disinformation Index (GDI) have increasingly called attention to how misinformation sites game ad tech^6,34^. Finally, our work features Facebook venues and domains that are primarily written in English. It would be valuable to evaluate whether our findings hold for domains using other languages.

## Methods

### Data

In November 2020, we identified vaccine-related Facebook groups/pages as described in^27^ based on whether they mentioned vaccine-related terms (“vaccine”, “vaxx”, “vaccines”, “jab”, etc.)—at least once. We excluded posts with keywords associated with animal vaccines - (“dog”, “cat”, “livestock”) to ensure that we only retained venues that routinely discussed human vaccines. We further narrowed down our list by retaining only those venues for which at least 20% of posts retrieved contained the substring “vacc” or “vax” or whose name contained at least one of the strings—vacc, vax, or jab. We were left with 451 groups/pages. These groups/pages were manually annotated as either pro-vaccine, anti-vaccine, or other. (Cohen’s k = 0.88, 95% CI 0.85–0.92).

From the 451 groups/pages, we extracted 46,472 unique URLs shared in their posts. These URLs belonged to 2436 distinct domains. Since the Facebook data collection overlaps with a period of frequent crackdowns on false vaccine information websites and potential ‘link rot’, many of the original URLs were removed or deleted by the page author. However, the underlying domains are more durable than the individual URLs. To afford stability to our measurements, instead of looking at the shared URLs directly, we examined the 2436 domains shared in the venues. For each domain, we extracted the content of each article posted between November 2019 and August 2021, resulting in 412,168 URLs.

We collected the HTML source code of the entire set of 412,168 URLs. For HTML measures (Ad count and Donation count), we analyzed the full set of URLs. Because Ad Occupancy and Outbound Links are calculated on live versions of the site and are more computationally expensive, we performed a stratified sampling technique to prune our list. We randomly sampled 15 URLs per domain. Some of the links that were sampled were dead, leaving us with 30,816 URLs for the Outbound link calculation. Similar page-take downs and additional link rot left us with 29,713 links for the Ad Occupancy calculations. See Table 2 for detailed information about the number of URLs analyzed to create each measure.

**Table 2:** Descriptive information for vaccine-related Facebook venues.

Venues	
Pro-vaccine venues	190
Anti-vaccine venues	261

Domains	
Ad count	2435
Donation count	2435
Ad occupancy	2390
Outbound links	2380

URLs	
Ad count	412,168
Donation count	412,168
Ad occupancy	29,713
Outbound links	30,816

The number of Facebook venues, domains, and URLs used to calculate our monetization measures. Facebook venues from^27^.

### Measures

Our first measure of the monetization intensity of a website is the ratio of ad space to text content on the page, which we call Ad Occupancy. Because page space is finite, space dedicated to ads comes at the cost of providing informative content and thus reflects, all else equal, a desire to drive revenue vs. conveying information.

**Figure 5:**
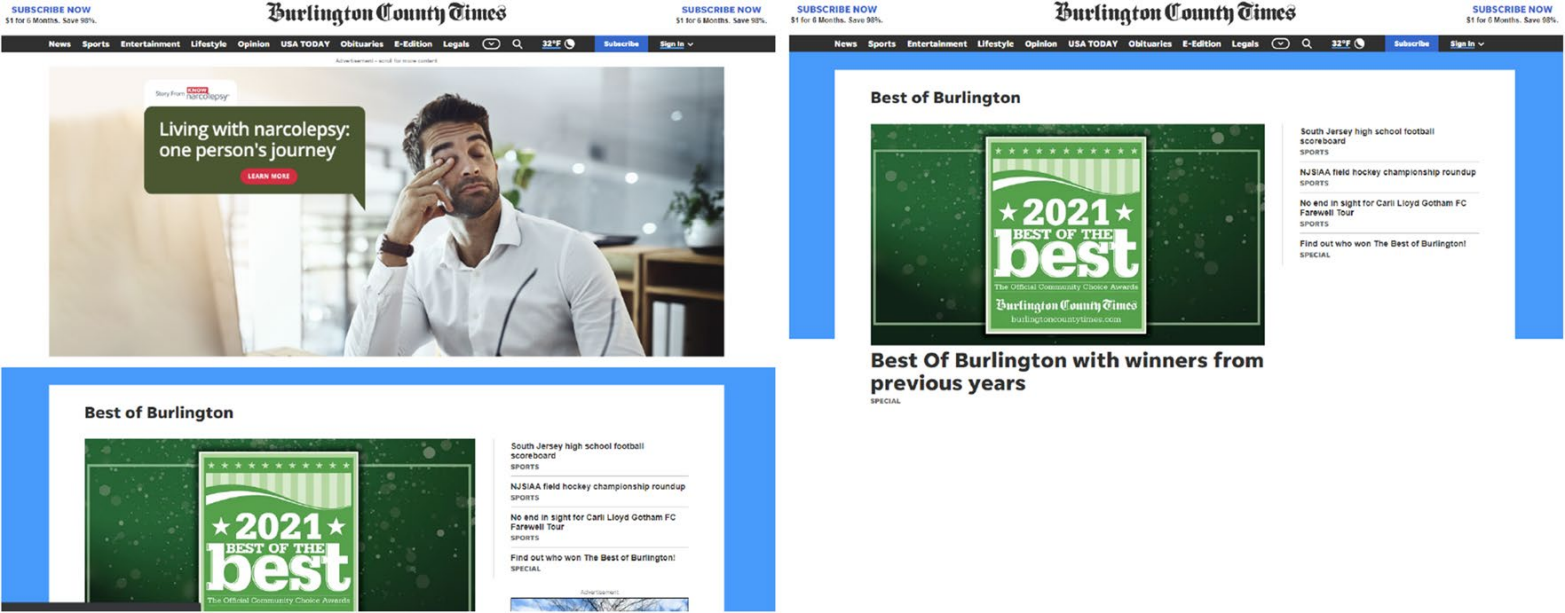
An example of ad occupancy. A web page rendered with (left) and without advertisements (right).

To quantify the measure, we render each page twice using Selenium—which provides a collection of language-specific bindings to drive a browser—Google Chrome in our case. In the first pass, we let the Selenium WebDriver access the URL uninhibited (Fig. 5). Once rendered, we capture each element rendered by its XPath and calculate its width and height, and thereby the area of the element, where XPath is a syntax for finding elements on web pages. Selenium provides a robust solution to estimate the size of web pages because it locates elements that are not found by ID, class, or name locators. The area of the rendered webpage is the sum of all element areas. Note that calculating area in this manner accounts for the size of pop-up ads, as well as banner ads and other inline display ads. In the second pass, we load the AdBlocker v4.35.0 Chrome extension to our Selenium Webdriver and render the same page again. AdBlocker removes ad elements including pop-ups and banner ads. We again calculate the area of the webpage as the sum of the areas of all the individual elements rendered. The differences in areas of the site rendered with and without ads allow us to estimate the percentage of each page dedicated to ads.

Ad Occupancy is then calculated as:

1 $$\begin{aligned} \text {Ad \, Occupancy} = \frac{\text {Area (Page \, w/ads) - Area (Page \, w/o \, ads)}}{\text {Area (Page \, w/ads)}}*100 \end{aligned}$$

Our second measure counts the number of distinct ads on a webpage. A page might dedicate less overall space to ads, but embed multiple distinct ads to monetize its user base. Doing so would lead to a cluttered and unattractive user experience, again trading potential revenue for the opportunity to convey meaningful information.

**Figure 6:**
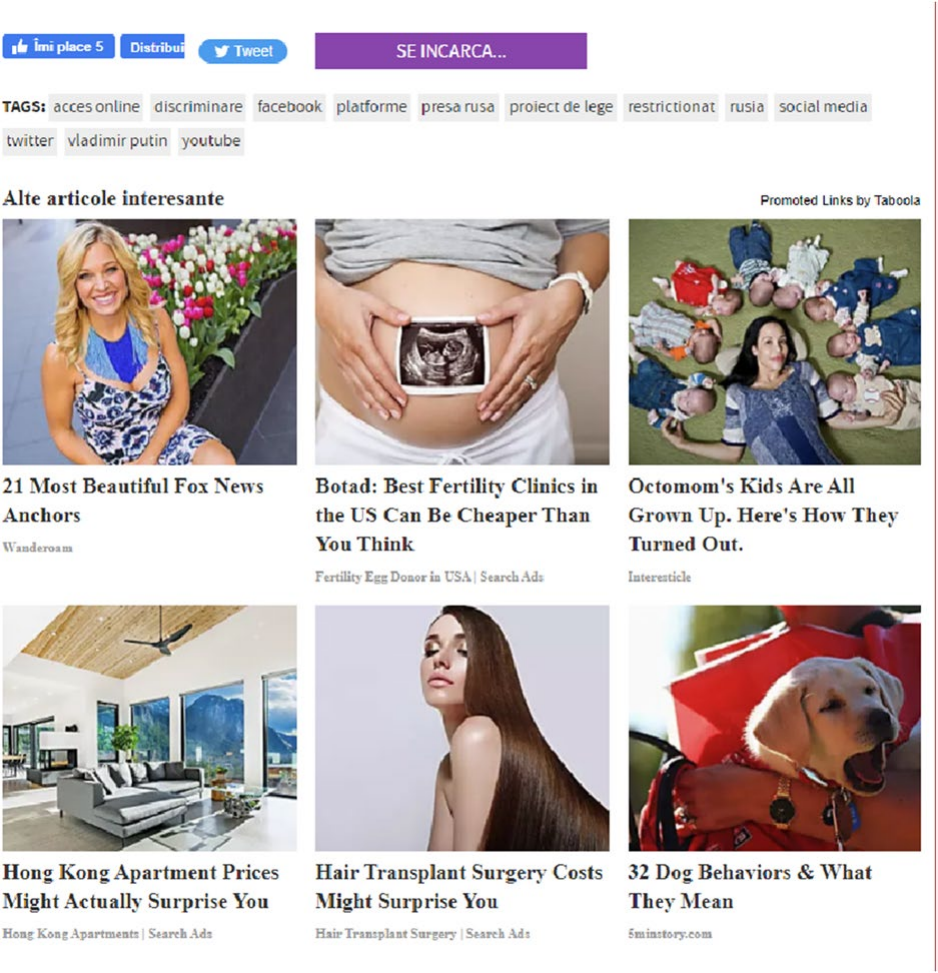
An example of ad count. A set of ads captured through div container counts.

Our measure is calculated by counting the number of div containers that contain the word ’ad’ in their field. For each site, we perform multiple crawls of each domain and take the median value. An example of the type of ads captured using this approach is found in Fig. 6.

**Figure 7:**
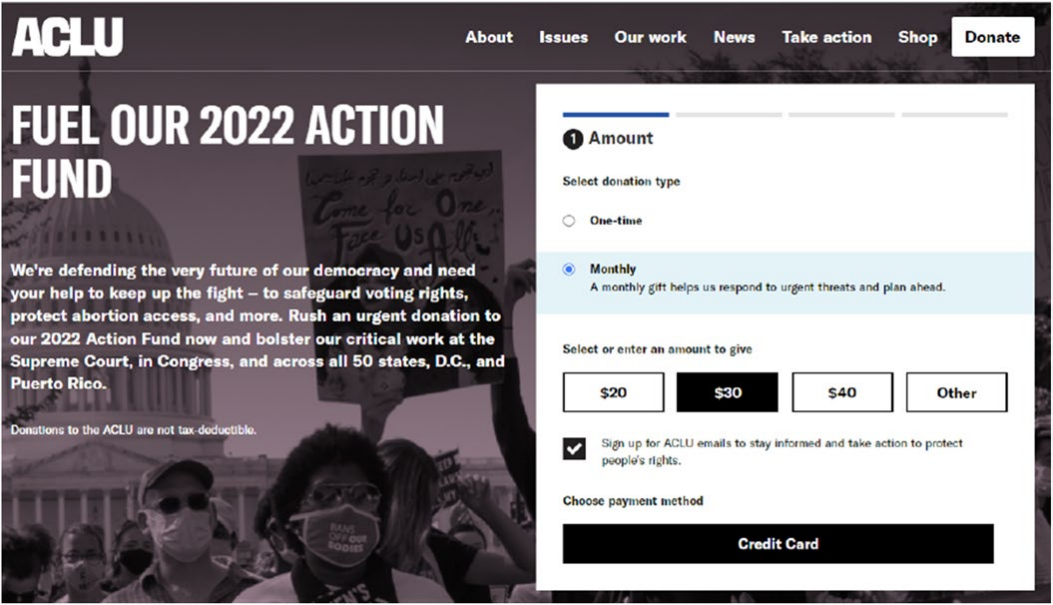
An example of a solicitation for donations from a webpage.

Our third measure captures sites attempting to collect donations from users. To measure the degree of solicitation of donations, we count the number of times the word “donate” occurs on the HTML of a page. To aggregate at the domain level, we calculate the median of the total number of ‘donate’ tags per URL for each domain. An example of the type of monetization method is found in Fig. 7.

Our fourth measure is a link-based measure—outbound links. Outbound links are point-of-reference dependent links from one website (host) to another. They are often used within the content of the host to add context to the piece by directing the reader to another source that complements it by adding or explaining important information related to the topic at hand. Since many domains earn income per click, such outbound linking is a clear source of revenue for the host website.

We scrape the source of each URL and extract links from the body of the webpage. After all the links from the body have been extracted, we remove the internal references—those that reference another page of the same domain, those that refer to the homepage, javascript links, and social media links for the domain. A count of the remaining number of links gives us the count of the outbound links.

To offer a better understanding of our measures, we present the results for some commonly encountered websites in Table 3. As we might expect, the website for the U.S. Food and Drug Administration has no evidence of monetization through ads or donations. The most heavily monetized site is *Huffpost*, a left-leaning news site. Interestingly, across most measures, the anti-vaccine site *Vaccine Impact* is less monetized than the *New York Times*.

**Table 3:** Site level monetization measures across five example domains.

Website type	Domain	Ads	Donations	Ad occ.	Outbound
Anti-vax	Vaccine impact	7	0	1.90	92.50
Gov	FDA	0	0	0	60.50
News	Huffpost	121.50	0	28.83	36
News	NY times	8	0	4.25	34.50
Business	Overleaf	0	0	0.003	19

All measures are the site-level median values.

### Statistical information

All comparisons were made using two-tailed Mann–Whitney *U* tests. This non-parametric test was chosen because the data did not meet the assumptions of normality and equal variances required for parametric tests.

## Data Availability

The data that support the findings of this study are available from CrowdTangle for Academics and Researchers, a third-party data provider owned and operated by Facebook but restrictions apply to the availability of these data, which were used under license for the current study, and so are not publicly available. CrowdTangle list IDs are provided in the references. Anyone with a CrowdTangle account may access these lists and the corresponding raw data. CrowdTangle’s terms of service prohibit providing raw data to anyone outside of a CrowdTangle user’s account. The user can share the findings, but not the data. If a journal asks for data to verify findings, the CrowdTangle user may send a .csv, but it cannot be posted publicly, and the journal must delete it after verification. Data are however available from the authors upon reasonable request to the corresponding author (broniatowski@gwu.edu), and with permission of CrowdTangle.

## Code availability

The code to reproduce all results reported in the manuscript will be made publicly available upon publication.
